# Recent advances in the study of prolamin storage protein organization and function

**DOI:** 10.3389/fpls.2014.00276

**Published:** 2014-06-20

**Authors:** David R. Holding

**Affiliations:** Department of Agronomy and Horticulture, Center for Plant Science Innovation, University of Nebraska-LincolnLincoln, NE, USA

**Keywords:** zein, kafirin, prolamin, storage_protein, endosperm, QPM, protein_body, deletion_mutagenesis

## Abstract

Prolamin storage proteins are the main repository for nitrogen in the endosperm of cereal seeds. These stable proteins accumulate at massive levels due to the high level expression from extensively duplicated genes in endoreduplicated cells. Such abundant accumulation is achieved through efficient packaging in endoplasmic reticulum localized protein bodies in a process that is not completely understood. Prolamins are also a key determinant of hard kernel texture in the mature seed; an essential characteristic of cereal grains like maize. However, deficiencies of key essential amino acids in prolamins result in relatively poor grain protein quality. The inverse relationship between prolamin accumulation and protein quality has fueled an interest in understanding the role of prolamins and other proteins in endosperm maturation. This article reviews recent technological advances that have enabled dissection of overlapping and non-redundant roles of prolamins, particularly the maize zeins. This has come through molecular characterization of mutants first identified many decades ago, selective down-regulation of specific zein genes or entire zein gene families, and most recently through combining deletion mutagenesis with current methods in genome and transcriptome profiling. Works aimed at understanding prolamin deposition and function as well as creating novel variants with improved nutritional and digestibility characteristics, are reported.

## INTRODUCTION

Although prolamins are the dominant class of seed storage protein in many cereals, this article illustrates their function and organization in maize and sorghum, the first and fifth most globally important cereal crops. Maize and sorghum are physically distinct in terms of their vegetative and reproductive architecture with maize having separate male and female reproductive organs and sorghum having hermaphroditic flowers that produce seed less than one tenth of the size of domestic maize seed. Sorghum is also usually more water use efficient and has potential for increased cultivation in marginal lands for this reason. Despite these differences, maize and sorghum are genetically more closely related than to other grasses. This is best shown by the phylogenetic relationships between their zein and kafirin prolamin-encoding genes (Xu and Messing, 2008).

## PROLAMIN GENES

Prolamins were initially distinguished as a group of proteins soluble in 70% ethanol (Osborne, 1897). However, differences in aqueous solubility and ability to form disulfide interactions, were later used to classify prolamin sub-families. The zeins are grouped into α, β, γ, and δ types based on these properties (Esen, 1987; Coleman and Larkins, 1999). Similarly, the kafirins are grouped into α, β, γ, and δ types based on their molecular weight, solubility, and gene sequence (Shull et al., 1991). Alpha zeins are encoded by four different gene sub-families (Z1A, Z1B, Z1C, and Z1D) that in the B73 reference line contain more than 40 genes in six chromosomal locations (Feng et al., 2009). There is, however, copy number and expression variation across different maize backgrounds. While substantial α-kafirin gene duplication also occurred in sorghum, all 20 α-kafirin genes are clustered at one chromosome 5 location in the BTx623 genome (Xu and Messing, 2008).

Alpha prolamins resolve at ~19- and 22-kDa on SDS-PAGE gels in both maize and sorghum. In maize, the 19-kDa α-zeins are encoded by the Z1A, Z1B, and Z1D subfamilies while the 22-kDa α-zeins are encodes by the Z1C subfamily (Song et al., 2001; Song and Messing, 2002). In sorghum, 19-kD α-kafirins are encoded by the K1α19 subfamily while the 22-kD α-kafirins are encoded by the K1α22 subfamily (Xu and Messing, 2009). Alpha prolamins cluster in a broad phylogenetic group (Group 1) as do the δ-zein genes. 10-kD and 18-kD δ-zeins are encoded by z2δ10 and z2δ18 genes in maize and k2δ2 and k2δ18 genes in sorghum (Xu and Messing, 2008, 2009). γ- and β-prolamins cluster within Group 2 (Xu and Messing, 2008, 2009). Unlike α-prolamins, Group 2 prolamin genes exist as single copies rather than highly duplicated gene families. In maize, this group consists of z2γ16 and z2γ27, encoding 16- and 27-kD γ-zeins, and z2γ50, encoding a 50-kD γ-zein. Similarly, k2γ27 and k2γ50 encode 27- and 50-kD γ-kafirins in sorghum although there is no 16-kD γ-kafirin. The maize z2γ16 gene is thought to derive from an unequal crossing-over event that occurred after allotetraploidization (Xu and Messing, 2008). Maize and sorghum also have genes encoding a 15-kD β-prolamin (z2β15 and k2β15) which are related to the γ-prolamins within Group 2 (Xu and Messing, 2008).

## PROLAMIN ACCUMULATION, THEIR EFFECT ON GRAIN TEXTURE, FUNCTIONALITY, AND PROTEIN QUALITY

**FIGURE 1:**
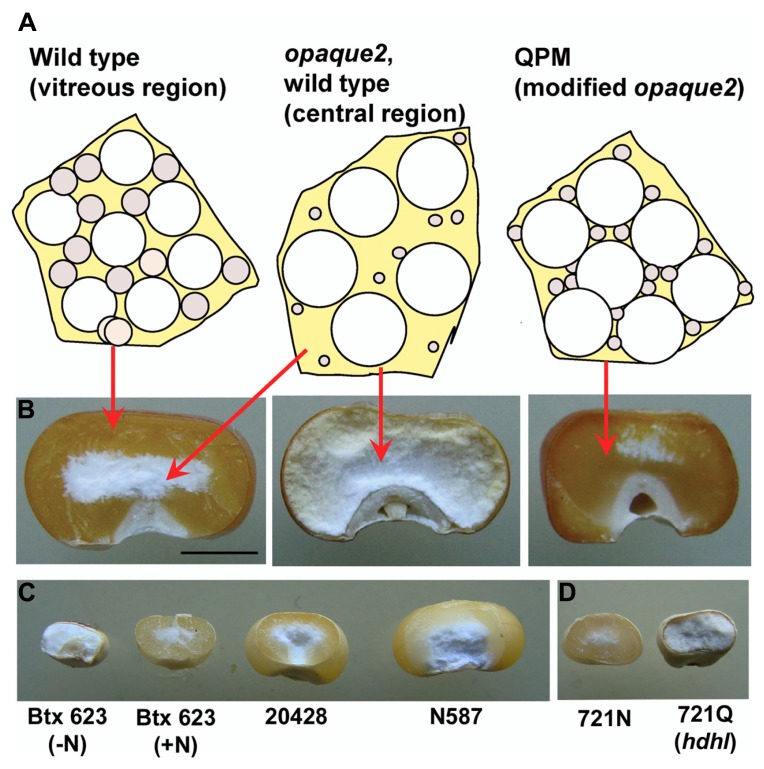
**Vitreous endosperm formation in maize and sorghum kernels. (A)** Individual cells of developing endosperm are represented with the relative size and abundance of starch grains (white spheres) and zein protein bodies (gray spheres) that are thought to result in vitreous or opaque endosperm in normal as well as in *opaque2* and modified *opaque2* (QPM) kernels. **(B)** Mature kernels of wild type, *opaque-2* and QPM cracked in half to reveal extent of vitreous endosperm. **(C)** Mature sorghum kernels cracked as in **B** to reveal vitreous endosperm and size variability in sorghum grain. **(D)** High digestibility high lysine (hdhl) sorghum mutant and its wild type isoline. Scale bar in **B** is 3 mm and refers to kernels in panels **B**–**D**.

Sorghum kernels are usually much smaller than maize kernels and both, but especially sorghum, show considerable heterogeneity in seed size across varieties (**Figure 1**). Despite this variability, maize and sorghum seed have similar endosperm composition having a high proportion of glassy or vitreous endosperm at the periphery of the mature kernel and a central opaque region (**Figure 1**). Vitreous endosperm is important for resistance to insect and fungal damage, resilience during harvest and storage, and many end use characteristics. Although we are still learning how vitreous endosperm is formed during kernel maruration, considerable evidence suggests that accumulation and packaging of prolamins into endoplasmic reticulum (ER) protein bodies play a central role. For example, in maize the vitreous outer region of the endosperm contains much more zein than the soft, opaque interior, and environmental conditions that cause reduced zein synthesis, such as nitrogen depletion, result in kernels that are soft and starchy throughout (Tsai et al., 1978). Sorghum kernels grown in limited nitrogen conditions are smaller and lack vitreous endosperm (**Figure 1**). Duvick (1961) proposed that in the periphery of the developing starchy endosperm, there is a certain ratio of starch grains, protein bodies, and viscous cytoplasm which dries down to form a rigid glass-like structure at kernel maturity (the vitreous endosperm; **Figure 1**). Toward the center of the endosperm, where zein protein bodies are smaller and less abundant, the rigid matrix is not formed during kernel desiccation, which results in the formation of the friable, opaque kernel center (**Figure 1**; Duvick, 1961). In opaque mutants, the central opaque region extends to the periphery of the endosperm (**Figure 1**).

Protein body formation in maize is controlled at several levels, including the temporal and spatial regulation of zein gene expression, the level of transcription and interactions that occur between the different types of zein proteins (Woo et al., 2001; Kim et al., 2002). Zeins are devoid of the essential amino acids, lysine and tryptophan (Mertz et al., 1964), but account for more than 70% of maize endosperm protein. This results in an overall protein content that is especially deficient in these amino acids. The equally dominant sorghum kafirins, share this nutritional deficiency, but it is compounded by the their poor digestibility (Aboubacar et al., 2001) that results from their high degree of disufide cross-linking.

**FIGURE 2:**
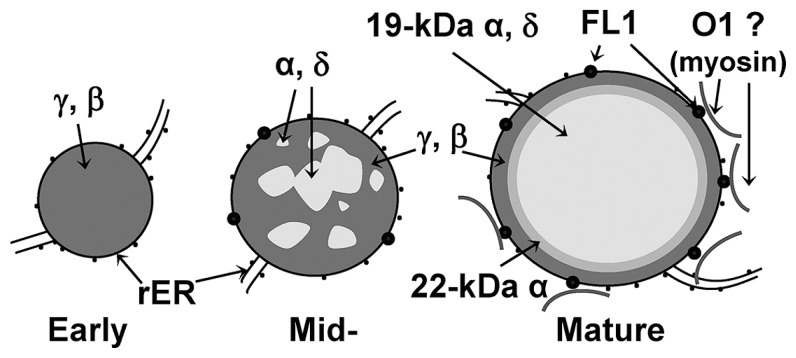
**Diagram of zein distribution in early, mid- and mature protein bodies**. Small black dots in membrane represent ribosomes while large black dots represent FLOURY1 protein. Curved lines outside protein bodies represent possible direct or indirect interaction with myosin.

Our knowledge of how prolamins are packaged at such high levels comes largely from maize. Zeins are retained as discretely layered membrane bound accretions in the ER (Lending and Larkins, 1989; **Figure 2**). Protein bodies start as small accretions consisting entirely of γ-zein, consistent with the slightly earlier onset of γ-zein gene expression (Woo et al., 2001). As protein bodies expand, α- and δ-zeins are sequestered into the protein body core, where they become encapsulated in a shell of γ-zeins. The 19-kD α-zeins are the most abundant class and immunological evidence suggests that the 22-kD α-zeins form an intermediary layer between the central 19-kD α-zeins and the γ-zein periphery (Holding et al., 2007). Although the γ-zeins have some functional redundancy, selective down-regulation has suggested they also have specialized roles as described below (Guo et al., 2013).

## MUTATIONS IN PROLAMIN GENES AND RELATED FACTORS SHED LIGHT ON PROLAMIN FUNCTIONAL ORGANIZATION

Natural and engineered mutants exhibiting reduced kernel hardness offer the opportunity to dissect the various biochemical and biophysical processes that affect vitreous endosperm formation, and consequently their study is of significant agronomic importance. Kernels of these mutants are opaque since they do not transmit light and often show defects in the accumulation of zeins or their packaging into ER-localized protein bodies. However, it is now clear that other factors are also important determinants of kernel texture since several recent studies have shown that vitreous endosperm can be disrupted by processes that do not affect zein synthesis and protein body structure. For example, vitreous endosperm formation is abolished in the *floury1* mutant as a result of knocking out a protein body ER membrane protein which seems to be involved zein organization (Holding et al., 2007). Therefore, it is likely that further protein body-related organizational factors remain to be identified. Other opaque mutants are the result of genetic aberrations in processes unrelated to protein body formation such as amino acid biosynthesis, plastid development and cytoskeletal function (Holding et al., 2010; Myers et al., 2011; Wang et al., 2012). This indicates that further functional genomics is needed to generate a more complete understanding of the factors which control late endosperm development.

The most well-known of the maize opaque mutants is *opaque2* (*o2*) which has been widely studied because of the increased lysine and tryptophan accumulation (Mertz et al., 1964) resulting from its reduced accumulation of alpha zeins. Cloning of the *O2* gene revealed that it encodes a transcription factor that regulates α-zeins (Schmidt et al., 1990) as well as other genes such as pyruvate Pi dikinase (Maddaloni et al., 1996). Although the soft kernels and yield penalty of *o2* prevented its commercial success, subsequent breeding projects, including those in Mexico (Vasal et al., 1980) and South Africa (Geevers and Lake, 1992), led to the development of hard kernel *o2* varieties called quality protein maize (QPM). QPM kernels maintain the low levels of α-zeins and thus, retain the high levels of lysine and tryptophan but the genetic basis of *o2* endosperm modification is complicated and poorly understood. The most prominent biochemical feature of QPM endosperm is the accumulation of the 27-kD γ-zein at 2–3 fold higher levels than in wild type and *o2* (Wallace et al., 1990; Geetha et al., 1991; Lopes and Larkins, 1991). Although the genetic or epigenetic mechanism of this increase is unknown, the degree of QPM endosperm vitreousness closely correlates with the level of 27-kD γ-zein protein (Lopes and Larkins, 1991). Furthermore, the 27-kD γ-zein gene maps to the most significant QTL for endosperm modification in QPM located on chromosome 7 (Lopes and Larkins, 1995; Lopes et al., 1995; Holding et al., 2008, 2011). QPM endosperm accumulates larger numbers of small, γ-zein rich protein bodies (**Figure 1**) which are proposed to allow the formation of a rigid glassy matrix similar in texture to mature wild type endosperm (**Figure 1**). γ-zein is known to be essential for endosperm modification in QPM (Wu et al., 2010), although the extent to which it is alone sufficient is unknown.

Although the functional redundancy resulting from the multi-member α-zein gene families has prevented the identification of recessive mutants, several dominant opaque mutants have been characterized. The phenotypes in these mutants result from the accumulation of defective prolamins that interfere with normal prolamin deposition and cause ER stress responses (Coleman et al., 1997; Kim et al., 2004, 2006; Wu et al., 2013). Floury-2 (*fl2*), *Defective endosperm B30* (*De-B30*), and *Mucronate* (*Mc*) are caused by dominantly acting mutations in zein genes. *fl2*, *De-B30*, and *Mc* show pleiotropic effects and result in a general reduction of all zeins (causing increases in lysine-containing proteins as in *o2*) and lobed protein bodies (Lending and Larkins, 1992). In the case of *fl2* and *De-B30*, this result from mutations that cause the signal peptides to remain attached in the 22-kD α-zein and 19-kD α-zein respectively, resulting in aggregation of these proteins at the ER membrane (Gillikin et al., 1997; Kim et al., 2004). The *Mc* mutant (Soave and Salamini, 1984) results from a 38 bp deletion that leads to a frame-shift mutation in the 16-kD γ-zein (Kim et al., 2006). The abnormal zeins produced in these mutants result in ER stress and cause a constitutive unfolded protein response (UPR), as shown by the dramatic up-regulation of a number of UPR-associated genes (Hunter et al., 2002). In fact, elevated markers for endosperm stress is a common feature of all opaque mutants studied irrespective of discernible changes in zeins and zein protein bodies (Hunter et al., 2002). This leads to the suggestion that endosperm stress and a resulting energy crisis may be at least partially responsible for disrupting vitreous endosperm formation (Guo et al., 2012).

Zein and kafirin proteins are packaged into protein bodies that are inherently recalcitrant to digestion. This results from the disulfide cross-linked nature of the γ-prolamins themselves and the fact that that they form a shell of relatively low surface area in relation to the amount of prolamin packaged. The poor digestibility of prolamins is especially pronounced in sorghum. An opaque kernel sorghum mutant was identified in an EMS mutagenized population that had increased lysine content as a result of reduced kafirin accumulation, and most notably, a marked increase in protein digestibility (Oria et al., 2000). Called the “high digestibility high lysine” (*hdhl*) variant (**Figure 1**), this mutant has significant potential to improve the utility of sorghum as a human staple and livestock feed. The increased digestibility apparently results from increased protease accessibility caused by reticulation of kafirin protein body shape, in a manner reminiscent of *fl2*, *De-B30*, and *Mc* in maize. Furthermore, developing *hdhl* kernels also exhibit a defined UPR. These phenotypic similarities to the maize mutants prompted a directed cloning approach in which the mutation was first mapped to an alpha kafirin gene cluster and extensive genomic and cDNA sequencing identified a mutant-specific α-kafirin copy harboring point mutation (Wu et al., 2013). The mutation causes a threonine substitution of an alanine residue, that is strictly conserved at position 21 of the signal peptide of all known α-prolamins (Wu et al., 2013). This substitution causes a dominant-negative response through low level accumulation of an uncleaved α-kafirin (Wu et al., 2013).

The characterization of opaque mutants has shown that vitreous endosperm formation depends on the correct expression and processing of prolamins themselves but also on factors that may have indirect roles in prolamin protein bodies such as Floury-1 and *Opaque-1* (Holding et al., 2007; Wang et al., 2012). Floury-1 was identified as a protein body ER membrane-specific protein, through a *Mutator* knock-out line that displays normal amounts of zein proteins and normal protein body size and shape, but slightly disorganized zein organization (Holding et al., 2007). Floury-1 contains a domain of unknown function (DUF593) for which the location inside or outside the ER lumen was not determined (Holding et al., 2007). Recently, screens for endomembrane proteins that bind myosin XI proteins in *Arabidopsis* identified myosin receptor proteins that bind myosin through DUF593 (Peremyslov et al., 2013). This suggests that Fl1 may function to attach protein bodies to the cytoskeleton and may explain the absence of a severe protein body phenotype in *fl1*. Similar to *fl1, o1* also does not have reduced zein accumulation but has a reduced number of slightly smaller protein bodies (Wang et al., 2012). *O1* was identified by positional cloning and encodes a myosin XI protein that is associated with cisternal and protein body ER (Wang et al., 2012). Although it has not been demonstrated, this suggests there may be a direct or indirect functional interaction between FL1 and O1 (**Figure 2**) and may suggest that the cytoskeleton plays an essential role in endosperm maturation.

Factors unrelated to prolamins are also essential for vitreous endosperm formation as demonstrated by opaque mutants such as *mto140*, *o7*, and *o5* (Holding et al., 2010; Miclaus et al., 2011a; Myers et al., 2011). *MTO140* encodes a member of the maize arogenate dehydrogenase family that are involved in tyrosine biosynthesis (Holding et al., 2010), while *O7* encodes an acyl-CoA synthetase-like protein (Miclaus et al., 2011a). *O5* encodes the major biosynthetic enzyme for synthesis of chloroplast membrane lipids, monogalactosyldiacylglycerol synthase and the mutant is specifically defective in galactolipids necessary for amyloplast and chloroplast function (Myers et al., 2011). As described in the section on deletion mutagenesis below, kernel opacity is a pleiotropic characteristic of kernel mutants also displaying other phenotypes such as small kernel, rough kernel, defective kernel, viviparary, and partial empty pericarp.

## TRANSGENIC EFFORTS TO OFFSET AMINO ACID DEFICIENCIES IN MAIZE

Various types of biotechnological approaches have been considered for improving the amino acid composition of maize (Holding and Larkins, 2008). In order to increase the methionine content, Lai and Messing (2002) created transgenic maize plants expressing a chimeric gene consisting of the coding region of 10-kD δ-zein and the promoter and 5′ untranslated region of the 27-kDa γ-zein. Although the effects on synthesis of endogenous high-sulfur zeins were not reported, uniformly high levels of 10-kDa δ-zein and methionine were observed and maintained over five backcross generations. Initial poultry feeding studies suggested that the transgenic grain was as effective as non-transgenic grain supplemented with free methionine.

For increasing the lysine content in maize, either non-maize, lysine-rich proteins can be expressed in an endosperm specific manner, or the zein sequences themselves can be manipulated to contain lysine. The former approach has been tried with a number of proteins which have mostly be expressed using γ- or α-zein promoters (Kriz, 2009). In order to make a significant difference to mature kernel lysine content, transgenic proteins must be driven to accumulate in very high amounts, in forms that do not interfere with the normal timing and pattern of endosperm programmed cell death and in a manner that does not induce UPR or kernel opacity. Furthermore, candidate proteins must meet stringent standards for potential allergenicity.

For the above reasons, perhaps the most promising way to elevate endosperm lysine is by modifying the coding sequences of the zein genes themselves. Lysine-containing zeins are more likely to be stored in their native ER protein body form and potentially in high enough quantities to significantly impact lysine levels. Preliminary studies in modifying a 19-kD α-zein with lysine residues, showed the transgenic protein to accumulate in protein body-like structures in *Xenopus* oocytes. Since the 19-kD α-zein is by far the most abundant zein, being packaged in the center of protein bodies (Holding et al., 2007), it may only be necessary to substitute a fraction of the native protein with a modified protein to make a significant impact on kernel protein quality. The 27-kD γ-zein, with its suspected role as the initiator of protein body formation, and as the major *o2* modifier, as well as its abundant accumulation, is also a good candidate for substituting certain amino acids with lysine. One study showed, using transient transformation of maize, that a 27-kD γ-zein in which (Pro-Lys)n sequences were inserted contiguous to or in substitution of the Pro-Xaa region, that the modified γ-zein co-localized with endogenous alpha- and gamma-zeins (Torrent et al., 1997). We are further investigating this type of approach using custom gene synthesis and the latest protein modeling programs that can assist in selection residues for substitution than are least likely to adversely affect normal zein packaging. To increase the chances of lysine-rich transgenic proteins being driven to accumulate at significant levels, it may be necessary to reduce the accumulation of native α-zeins. An effective way to do this is using RNA interference (RNAi) as described below.

Zeins are much less likely to invoke allergic reactions than the wheat prolamins, the glutens. However, when considering potential transgenic bio-fortification approaches, allergen databases must be consulted. Among several allergenic maize seed proteins, precursors of α- and β-zein have both been shown to be allergenic (Pastorello et al., 2009).

## RNA INTERFERENCE LINES HAVE ALLOWED DISSECTION OF REDUNDANT AND NON-REDUNDANT PROLAMIN FUNCTION

Naturally occurring and induced opaque mutants have been extensively studied because of their potential for grain nutritional improvement and have created an understanding of prolamin packaging in ER protein bodies and its relationship to ER protein quality control and UPR. However, opaque mutants carrying nutritional benefits such as *o2* and *fl2* also carry negative pleiotropic characteristics. Furthermore, the extensive gene duplication and gene redundancy, especially in the α-prolamin classes, have resulted in a lack of recessive prolamin mutants, and an inability to infer the relative prolamin functional redundancy and non-redundancy. RNAi has been an effective tool for further addressing maize and sorghum nutritional potential as well as providing new information regarding functions of specific prolamin classes (Holding and Messing, 2013).

**FIGURE 3:**
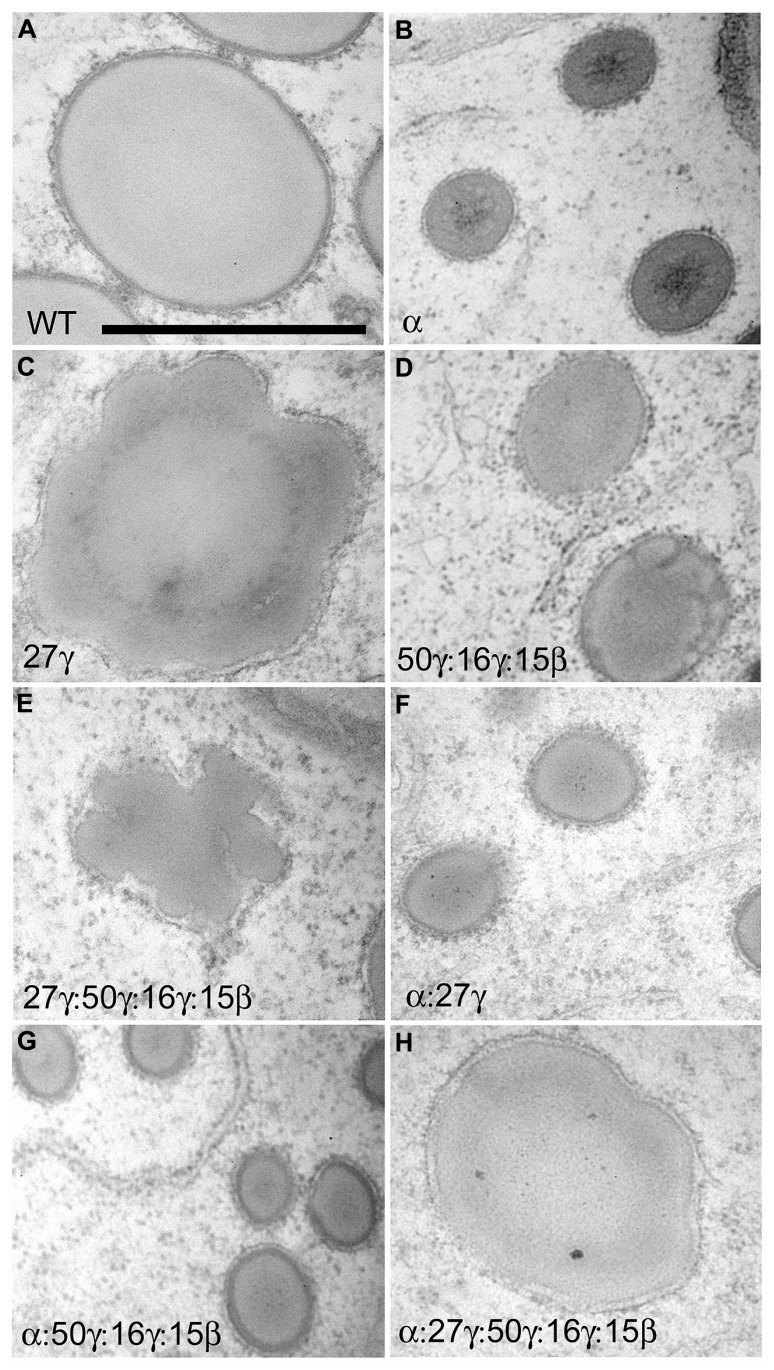
**TEM analysis showing protein body size and morphology in the fourth sub-aleurone starchy layer of 18 DAP endosperm in zein RNAi lines and their crosses**. Scale bar in **A** is 1 μm and refers to all panels. RNAi transgenes present are shown in bottom left of each panel in **A–H**. Figure copyright of American Society of Plant Biologists.

Initial use of RNAi to eliminate α-zeins revealed the possibility of creating dominant, non-pleiotropic low-zein lines for lysine improvement. The dominance of such transgenes circumvents one of the limitations of *o2* based varieties which is that the *o2* allele must be maintained in the homozygous mutant state, which is easily lost through wild type pollen contamination, and is especially problematic in an open pollinated QPM setting. Originally, the 22-kD α-zein was targeted and transgenic lines had considerably reduced α-zein and concomitant increase in lysine (Segal et al., 2003) despite lines accumulating substantial amounts of 19-kD α-zein. Protein bodies were of reduced size due to reduced α-zein filling and notably, exhibited distorted lobed appearances. Similarly, a study in sorghum aimed to increase lysine content and digestibility by removing α-kafirins (Kumar et al., 2012). Like the maize study, only one class of α-kafirins (22-kD) was targeted. Though a reduction in protein body size was not reported, the transgene resulted in protein body lobing similar to the 22-kD α-zein RNAi lines and dominant α-zein signal peptide mutants (Kumar et al., 2012). This suggests that 22-kD α-prolamins may be essential for correct packaging of the 19-kD α-prolamins and is consistent with the observed peripheral location of the 22-kD α-zeins relative to the 19-kD α-zeins (Holding et al., 2007). Later works targeting both the 19- and 22-kD α-zeins did not address the morphological effects on protein bodies (Huang et al., 2006; Wu and Messing, 2011). Using a chimeric α-zein RNAi cassette, comprised of ~250 bp regions of the most abundantly expressed Z1A, Z1B, Z1C, and Z1D α-zein family members in B73, and the 27-kD γ-zein promoter and the cauliflower mosaic virus 35S terminator, we suppressed both 22- and 19-kD α-zeins to low levels. This resulted in very small protein bodies but did not suppress protein body number per unit area suggesting that while α-zeins drive protein body filling, they are not involved in protein body initiation (Guo et al., 2013; **Figure 3**). In contrast to suppression of 22-kD α-zein alone, no lobing or distortion of protein bodies was observed in support of the suggestion that such phenotypes are generated by inappropriately located or unconstrained α-zeins. Similarly, in *o2* where all α-zeins are reduced, protein bodies are small but not misshapen (Geetha et al., 1991).

Apart from the *Mc* mutant, which accumulates a dominant-negative 16-kD γ-zein with a nonsense C-terminus as a result of a frame-shift mutation (Kim et al., 2006), mutants of γ-prolamins have not been described. This may be partly due to some functional redundance of different γ-prolamins but knowledge of their roles is very limited aside from their ability to form the cross-linked outer shell of ER-protein bodies. A specialized role for the 27-kD γ-zein in protein body initiation has been inferred from its increase in QPM endosperm and concomitant increase in protein body number. This is also supported by the *o15* mutant in which reduced 27-kD γ-zein leads to reduced protein body number (Dannenhoffer et al., 1995).

RNA interference lines have provided functional insight about different γ-zeins. Transgenic events that targeted the 27-/16-kD γ-zeins, whose genes share sequence similarity, as well as a separate 15-kD β-zein RNAi event caused minor morphological changes to protein bodies (Wu and Messing, 2010). Although an incidence of very small protein bodies was shown in both cases, the majority of protein bodies were of normal size and no changes in protein body number were reported (Wu and Messing, 2010). The 50-kD γ-zein was not targeted in this study since it was assumed to have a minor role in protein body formation due to its low abundance. In fact, by manipulating the ethanolic extraction procedure, it has been shown that the 50-kD γ-zein has comparable abundance to other γ-zeins except the dominantly abundant 27-kD γ-zein (Guo et al., 2013).

We made RNAi constructs to dissect the role of 27-kD γ-zein with respect to other γ-zeins. The first was a complete 27-kD γ-zein gene RNAi (27-γ) driven by its native promoter and terminated by the cauliflower mosaic virus 35S terminator, and this almost completely inhibited synthesis of 27-kD γ-zein and, because of sequence similarity, significantly reduced 16-kD γ-zein. A second transgene was a synthetic RNAi gene consisting of ~250 bp regions of the 16- and 50-kD γ-zein and the 15-kD β-zein (16/50/15-γ) that were selected to have the least similarity to 27-kD γ-zein. In this case, the promoter of a dominantly expressed 22-kD α-zein gene was used in this case, to avoid potential co-suppressive effects on the 27-kD γ-zein level that could have resulted from using the 27-kD γ-zein promoter. The 16/50/15-γ transgene reduced all target proteins to very low levels while leaving 27-kD at high levels (Guo et al., 2013). Protein bodies in developing endosperm of 16/50/15-γ events were of normal shape but were of reduced size, and accumulated in normal numbers (**Figure 3**; Guo et al., 2013). This illustrates that while the 16/50/15-kD γ-zeins are necessary for protein body filling and α-zein encapsulation, they are not involved in protein body initiation. Conversely, the 27-γ RNAi did not reduce protein body size and induced undulations in protein body shape (**Figure 3**), suggesting that the 27-kD γ-zein is not necessary for the bulk of protein body filling and that other γ-zeins can fulfill this role. Most notably however, protein bodies in 27-γ events were of much lower number than control, suggesting that the 27-kD γ-zein alone has the role of protein body initiation (Guo et al., 2013). Combining the γ-zein transgenes resulted in addition of the reduced number/distortion phenotypes of the 27-γ RNAi with the reduced size phenotype of the 16/50/15-γ transgene (Guo et al., 2013; **Figure 3**). Similarly, combining either of the γ-zein RNAi transgenes with the α-zein RNAi resulted in small protein bodies (**Figure 3**) with the 27-γ transgene reducing protein body number more significantly than the 16/50/15-γ transgene (Guo et al., 2013). When all three transgenes were combined to reduce all zeins proportionately, while protein body number was very low, protein bodies had a normal size and relatively normal morphology (**Figure 3**; Guo et al., 2013). This showed that maintenance of an appropriate ratio of all zeins is critical for their proper storage.

## FUNCTIONAL GENOMICS OF MAIZE ENDOSPERM MATURATION USING DELETION MUTAGENESIS

**FIGURE 4:**
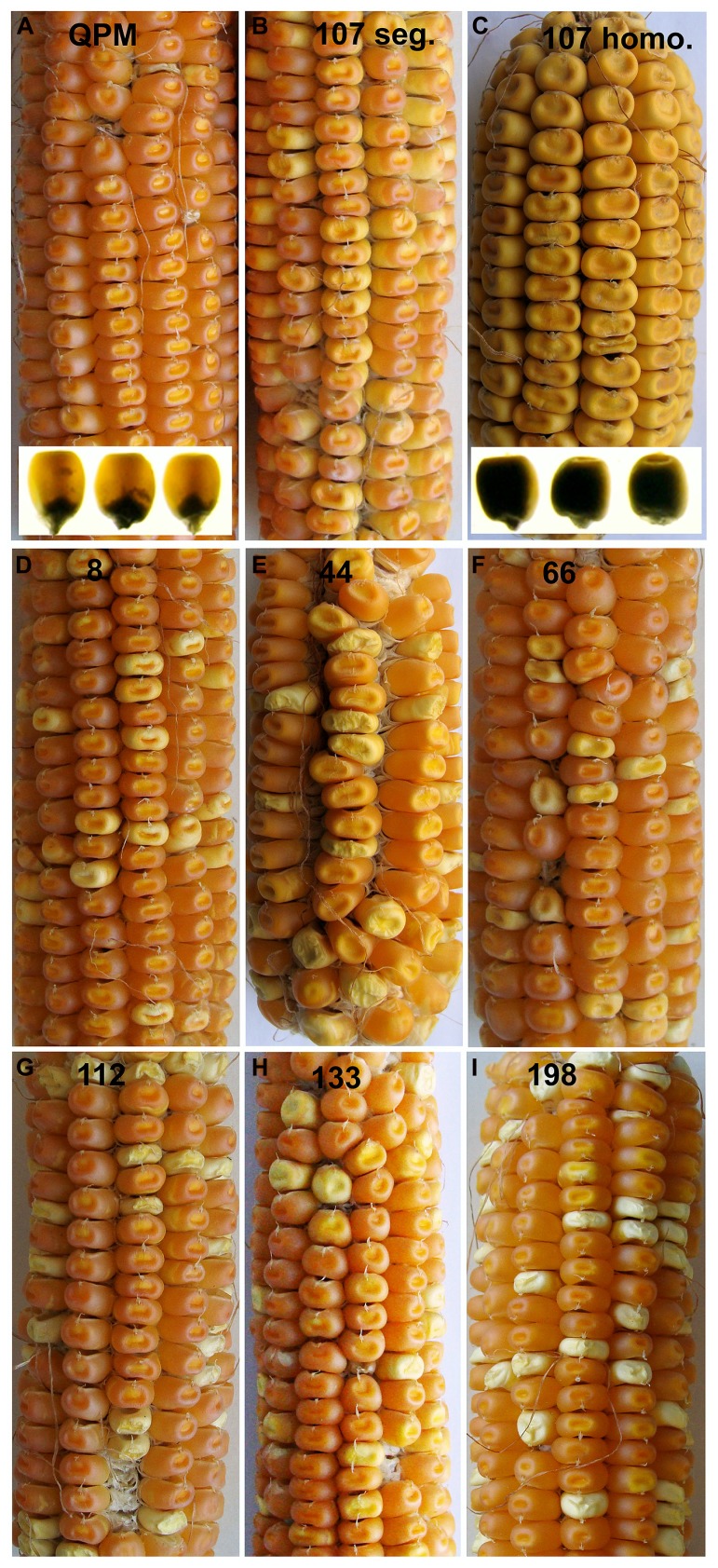
**M3 ear phenotypes of selected segregating K0326Y opaque deletion mutants**. K0326Y mutant line number is shown at the top center of each panel in **A–I**. Inserts in **A,C** show light box phenotypes. Modified from Yuan et al. (2014).

**FIGURE 5:**
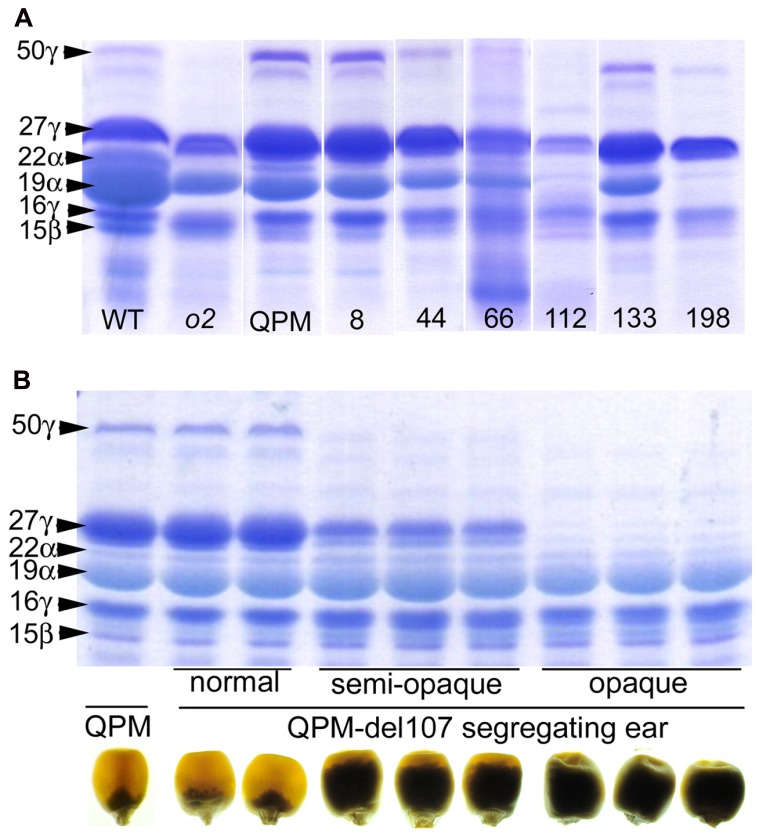
**(A)** SDS-PAGE zein profiles of selected K0326Y QPM opaque reversion mutants. **(B)** SDS-PAGE zein profiles and kernel vitreousness phenotypes of QPM, and hemizygous and homozygous line 107 gamma-zein deletion mutants. Modified from Yuan et al. (2014). Originals copyright of American Society of Plant Biologists.

While RNAi transgenes as well as different types of mutation often result in leaky expression, gene deletion mutagenesis, though random, has the advantage creating complete nulls. We investigated γ-irradiation for its potential to identify genome regions containing *o2* modifier genes in a QPM background. A small population of ~300 M3 families contained a number of recessive opaque revertant mutants (**Figure 4**) thus demonstrating its utility for identifying *o2* modifier genes as well as genes generally involved in kernel maturation (Yuan et al., 2014). This non-pleiotropic and non-lethal class of kernel mutants had varying effects on zein accumulation (**Figure 5**). The two most striking of these opaque mutants generated were unlike any previously described zein variants. The first of these, line 107, which has been thoroughly characterized at the molecular and phenotypic levels, is a null mutant of 27-kD γ-zein (Yuan et al., 2014). The second mutant, line 198, reduces 19-kD α-zeins to very low levels, in addition to the already low 22-kD α-zein caused by the *o2* mutation. Line 107 showed a complete absence of 27-kD γ-zein on SDS-PAGE gels, and the 50-kDa γ-zein was also undetectable. This was a preliminary indication that a deletion spanned both genes since they are known to be separated by only 27 kbp on chromosome 7 (Holding et al., 2008). Subsequent RT-PCR and PCR confirmed the absence transcripts and genes, while the unlinked 16-kD γ-zein was unaffected (Yuan et al., 2014). Illumina sequencing of exon-enriched genomic DNA showed that line 107 has a 1.2 Mbp deletion on chromosome 7.02 that includes both the 27- and 50-kD γ-zein genes. The increase in 27-kD γ-zein in QPM and its map position within the largest QTL for *o2* endosperm modification has long been known, but we also observed an increase in 50-kD γ-zein in QPM, possibly demonstrating a contribution to this QTL. The homozygous γ-zein deletion completely abolished endosperm modification since kernels were fully opaque. Interestingly, hemizygous kernels with a single copy of each gene accumulated intermediate amounts of both γ-zeins and were semi-modified (**Figure 5**), indicating a haploinsufficiency effect in which high level expression from both copies of these genes are necessary for vitreous endosperm formation in QPM. This unequivocally establishes 27-kD γ-zein as *o2* modifier gene. More recent work indicates that the haploinsufficiency of the 27-kD γ-zein does not apply to normal vitreous endosperm formation in a wild type (*O2*/*O2*) background, since kernels hemizygous for the 27-kD γ-zein deletion are fully vitreous.

The 19-kD α-zeins are encoded by genes within the Z1A1, Z1A2, Z1B, and Z1D families residing at four different loci (Miclaus et al., 2011b). Though the expression of all these genes is not equivalent within B73 and varies dramatically between genetic backgrounds (Feng et al., 2009), the very low abundance of 19-kD α-zein in QPM deletion line 198 endosperm is unlikely to result from a physical deletion at one of the four loci. Indeed, our data do not suggest a physical Z1 deletion in line 198. All Z1 classes including Z1C (22-kD α-zein) show substantially reduced transcript abundance compared with the non-mutagenized control. However, exon sequencing and RNA-seq did not reveal any physical Z1 gene deletions, cDNA sequencing did not identify missing species and bulked segregation analysis maps the mutation to a chromosome not containing Z1 loci. The more likely scenario is that the causative mutation in line 198 is within an *Opaque-2* unrelated gene with a direct or indirect role in regulating alpha-zein abundance. For other opaque mutants shown in **Figures 4 and 5**, mapping populations have been made and map positions are being used to guide prioritization of candidate gene deletions from exon-seq and RNA-seq data already generated.

Since even a small population of ~300 families in the QPM population yielded a more than 20 new opaque and small kernel mutants, we made a second mutagenized population in the B73 reference line. This population is much larger and resulted in 1793 M2 (second generation) ears. Rather than just *o2* modifier genes, the scope of this population is for more general seed functional genomics and direct manipulation of grain quality (such as by direct deletion of alpha zein sub-family loci). Since mutants are isogenic with the reference B73 genome, it will be considerably easier to assemble and utilize DNA and RNA-seq data when surveying the nature and extent of deletions within mutants.

The cells that give rise to the ear and the cells that give rise to the tassel are already specified and are physically separate in the embryonic maize shoot apical meristem (Poethig et al., 1986), and so a given mutation will not be present both ear and tassel of an M1 plant. Thus, M2 kernels will be hemizygous for such a mutation, and only show a kernel phenotype if it is dominant. Consequently, M3 families must be propagated to identify segregating recessive mutants. Among families already advanced to the M3 (10–15 plants each), there is substantial overlap between the seed phenotypes observed, since the majority of opaque mutants show some degree of reduced kernel size. Molecular genetic and biochemical characterization of these mutants will increase our understanding of the processes controlling kernel filling and its proper maturation. One priority is to identify mutants that have alterations in the ratio of zein and non-zein fractions and several mutants that have relatively increased amounts of non-zein proteins, with or without a corresponding decrease in zein proteins have been identified. F1 outcrosses are being made to Mo17 and subsequently F2 mapping populations will be used to map the causative mutations to a chromosome bin through Bulked Segregant Analysis. Map positions will be used to guide interpretation of Illumina HiSeq2500 DNA- and RNA-seg data as well as LC-MS/MS quantitative complete proteome profiling data.

## CONCLUDING REMARKS

Studies of opaque mutants, especially *opaque-2* and QPM, were fueled by the prospect of maize varieties with improved protein quality. Though QPM varieties have been bred and are in use in many developing countries, their potential has not been realized in the U.S. Furthermore, characterization of other opaque mutants, especially those that do not improve protein quality, has been slow until recently. Our knowledge of the mechanisms of protein body formation and the role of zein storage proteins and other unknown factors in vitreous endosperm formation in both wild type and QPM contexts, has been limited. However, over the last decade, several technological developments have accelerated our understanding of these processes. Mutator transposon induced opaque mutants led to the identification of several non-zein factors with roles in endosperm maturation. An enhanced ability to perform map-based cloning in maize has resulted in several very old, broadly mapped opaque mutants being cloned in recent years. RNAi studies have also shed light on the redundant and non-redundant roles of zeins in protein body formation. Deletion mutagenesis is emerging as an additional way to confirm suspected *opaque-2* modifier genes and potentially lead to identification of unknown ones. We are using this approach to generate new kernel mutants that will hasten seed functional genomics when paired with current genetic mapping resources, DNA- and RNA-sequencing capacities and proteomics.

## Conflict of Interest Statement

The Guest Associate Editor Brian A. Larkins declares that, despite being affiliated to the same institution as the author David R. Holding, the review process was handled objectively and no conflict of interest exists. The author declares that the research was conducted in the absence of any commercial or financial relationships that could be construed as a potential conflict of interest.

## References

[B1] Aboubacar A. Axtell J. D. Huang C. P. Hamaker B. R. (2001). A rapid protein digestibility assay for identifying highly digestible sorghum lines. *Cereal Chem.* 78 160–165 10.1094/Cchem.2001.78.2.160

[B2] Coleman C. E. Clore A. M. Ranch J. P. Higgins R. Lopes M. A. Larkins B. A. (1997). Expression of a mutant alpha-zein creates the floury2 phenotype in transgenic maize. *Proc. Natl. Acad. Sci. U.S.A.* 94 7094–7097 10.1073/pnas.94.13.7094 9192697 PMC21290

[B3] Coleman C. E. Larkins B. A. (1999). “The prolamins of maize,” in *Seed Proteins* edsShewry P. Casey R. (Dordrecht: Kluwer Academic Publishers) 109–139 10.1007/978-94-011-4431-5_6

[B4] Dannenhoffer J. M. Bostwick D. E. Or E. Larkins B. A. (1995). Opaque-15, a maize mutation with properties of a defective opaque-2 modifier. *Proc. Natl. Acad. Sci. U.S.A.* 92 1931–1935 10.1073/pnas.92.6.1931 7892202 PMC42396

[B5] Duvick D. N. (1961). Protein granules of maize endosperm cells. *Cereal Chem.* 38 374–385

[B6] Esen A. (1987). A proposed nomenclature for the alcohol-soluble proteins (zeins) of maize (*Zea-mays*-L). *J. Cereal Sci.* 5 117–128 10.1016/S0733-5210(87)80015-2

[B7] Feng L. N. Zhu J. Wang G. Tang Y. P. Chen H. J. Jin W. B. (2009). Expressional profiling study revealed unique expressional patterns and dramatic expressional divergence of maize alpha-zein super gene family. *Plant Mol. Biol.* 69 649–659 10.1007/s11103-008-9444-z 19112555

[B8] Geetha K. B. Lending C. R. Lopes M. A. Wallace J. C. Larkins B. A. (1991). Opaque-2 modifiers increase gamma-zein synthesis and alter its spatial-distribution in maize endosperm. *Plant Cell* 3 1207–1219 10.1105/tpc.3.11.1207 1821766 PMC160087

[B9] Geevers H. O. Lake J. K. (1992). “Development of modified opaque-2 maize in South Africa,” in *Quality Protein Maize* ed.Mertz E. T. (St. Paul, Minnesota: American Society of Cereal Chemists) 49–78

[B10] Gillikin J. W. Zhang F. Coleman C. E. Bass H. W. Larkins B. A. Boston R. S. (1997). A defective signal peptide tethers the floury-2 zein to the endoplasmic reticulum membrane. *Plant Physiol.* 114 345–352 10.1104/pp.114.1.345 9159955 PMC158310

[B11] Guo X. Ronhovde K. Yuan L. L. Yao B. Soundararajan M. P. Elthon T. (2012). Pyrophosphate-dependent fructose-6-phosphate 1-phosphotransferase induction and attenuation of hsp gene expression during endosperm modification in quality protein maize. *Plant Physiol.* 158 917–929 10.1104/pp.111.191163 22158678 PMC3271778

[B12] Guo X. Yuan L. Chen H. Sato S. J. Clemente T. E. Holding D. R. (2013). Nonredundant function of zeins and their correct stoichiometric ratio drive protein body formation in maize endosperm. *Plant Physiol.* 162 1359–1369 10.1104/pp.113.218941 23677936 PMC3707540

[B13] Holding D. R. Hunter B. G. Chung T. Gibbon B. C. Ford C. F. Bharti A. K. (2008). Genetic analysis of opaque2 modifier loci in quality protein maize. *Theor. Appl. Genet.* 117 157–170 10.1007/s00122-008-0762-y 18427771

[B14] Holding D. R. Hunter B. G. Klingler J. P. Wu S. Guo X. Gibbon B. C. (2011). Characterization of opaque2 modifier QTLs and candidate genes in recombinant inbred lines derived from the K0326Y quality protein maize inbred. *Theor. Appl. Gen.* 122 783–794 10.1007/s00122-010-1486-3 21076810

[B15] Holding D. R. Larkins B. A. (2008). Genetic engineering of seed storage proteins. *Bioeng. Mol. Biol. Plant Pathw.* 1 107–133 10.1016/S1755-0408(07)01005-3

[B16] Holding D. R. Meeley R. B. Hazebroek J. Selinger D. Gruis F. Jung R. (2010). Identification and characterization of the maize arogenate dehydrogenase gene family. *J. Exp. Bot.* 61 3663–3673 10.1093/Jxb/Erq179 20558569 PMC2921203

[B17] Holding D. R. Messing J. (2013). “Evolution, structure, and function of prolamin storage proteins,” in *Seed Genomics* ed.Becraft P. (New York: John Wiley & Sons) 139–158

[B18] Holding D. R. Otegui M. S. Li B. L. Meeley R. B. Dam T. Hunter B. G. (2007). The maize floury1 gene encodes a novel endoplasmic reticulum protein involved in zein protein body formation. *Plant Cell* 19 2569–2582 10.1105/tpc.107/053538 17693529 PMC2002605

[B19] Huang S. Frizzi A. Florida C. A. Kruger D. E. Luethy M. H. (2006). High lysine and high tryptophan transgenic maize resulting from the reduction of both 19- and 22-kD alpha-zeins. *Plant Mol. Biol.* 61 525–535 10.1007/s11103-006-0027-6 16830184

[B20] Hunter B. G. Beatty M. K. Singletary G. W. Hamaker B. R. Dilkes B. P. Larkins B. A. (2002). Maize opaque endosperm mutations create extensive changes in patterns of gene expression. *Plant Cell* 14 2591–2612 10.1105/tpc.003905 12368507 PMC151238

[B21] Kim C. S. Gibbon B. C. Gillikin J. W. Larkins B. A. Boston R. S. Jung R. (2006). The maize Mucronate mutation is a deletion in the 16-kDa gamma-zein gene that induces the unfolded protein response. *Plant J.* 48 440–451 10.1111/j.1365-313X.2006.02884.x 17010110

[B22] Kim C. S. Hunter B. G. Kraft J. Boston R. S. Yans S. Jung R. (2004). A defective signal peptide in a 19-kD alpha-zein protein causes the unfolded protein response and an opaque endosperm phenotype in the maize De*-B30 mutant. *Plant Physiol.* 134 380–387 10.1104/pp.103.031310 14657407 PMC316317

[B23] Kim C. S. Woo Y. M. Clore A. M. Burnett R. J. Carneiro N. P. Larkins B. A. (2002). Zein protein interactions, rather than the asymmetric distribution of zein mRNAs on endoplasmic reticulum membranes, influence protein body formation in maize endosperm. *Plant Cell* 14 655–672 10.1105/tpc.010431 11910012 PMC150587

[B24] Kriz A. L. (2009). “Enhancement of amino acid availability in corn grain,” in *Molecular Genetic Approaches to Maize Improvement* edsKriz A. L. Larkins B. A. (Heidelberg: Springer) 79–89 10.1007/978-3-540-68922-5

[B25] Kumar T. Dweikat I. Sato S. Ge Z. X. Nersesian N. Chen H. (2012). Modulation of kernel storage proteins in grain sorghum (*Sorghum bicolor* (L.) Moench). *Plant Biotechnol. J.* 10 533–544 10.1111/j.1467-7652.2012.00685.x 22353344

[B26] Lai J. S. Messing J. (2002). Increasing maize seed methionine by mRNA stability. *Plant J.* 30 395–402 10.1046/j.1365-313X.2001.01285.x 12028570

[B27] Lending C. R. Larkins B. A. (1989). Changes in the zein composition of protein bodies during maize endosperm development. *Plant Cell* 1 1011–1023 10.1105/tpc.1.10.1011 2562552 PMC159838

[B28] Lending C. R. Larkins B. A. (1992). Effect of the floury-2 locus on protein body formation during maize endosperm development. *Protoplasma* 171 123–133 10.1007/BF01403727

[B29] Lopes M. A. Larkins B. A. (1991). Gamma-zein content is related to endosperm modification in Quality Protein Maize. *Crop Sci.* 31 1655–1662 10.2135/cropsci1991.0011183X003100060055x

[B30] Lopes M. A. Larkins B. A. (1995). Genetic-analysis of opaque2 modifier gene activity in maize endosperm. *Theor. Appl. Gen.* 91 274–281 10.1007/BF00220889 24169775

[B31] Lopes M. A. Takasaki K. Bostwick D. E. Helentjaris T. Larkins B. A. (1995). Identification of opaque 2 modifier loci in quality protein maize. *Mol. Gen. Genet.* 247 603–613 10.1007/BF00290352 7603440

[B32] Maddaloni M. Donini G. Balconi C. Rizzi E. Gallusci P. Forlani F. (1996). The transcriptional activator Opaque-2 controls the expression of a cytosolic form of pyruvate orthophosphate dikinase-1 in maize endosperms. *Mol. Gen. Genet.* 250 647–654 10.1007/BF02174452 8676867

[B33] Mertz E. T. Nelson O. E. Bates L. S. (1964). Mutant gene that changes protein composition and increases lysine content of maize endosperm. *Science* 145 279–280 10.1126/science.145.3629.279 14171571

[B34] Miclaus M. Wu Y. R. Xu J. H. Dooner H. K. Messing J. (2011a). The maize high-lysine mutant opaque7 Is defective in an acyl-CoA synthetase-like protein. *Genetics* 189 1271–1280 10.1534/genetics.111.133918 21926304 PMC3241421

[B35] Miclaus M. Xu J. H. Messing J. (2011b). Differential gene expression and epiregulation of alpha zein gene copies in maize haplotypes. *PLoS genetics* 7:e1002131 10.1371/journal.pgen.1002131 PMC3121756 21731501

[B36] Myers A. M. James M. G. Lin Q. H. Yi G. Stinard P. S. Hennen-Bierwagen T. A. (2011). Maize opaque5 encodes monogalactosyldiacylglycerol synthase and specifically affects galactolipids necessary for amyloplast and chloroplast function. *Plant Cell* 23 2331–2347 10.1105/tpc.111.087205 21685260 PMC3160020

[B37] Oria M. P. Hamaker B. R. Axtell J. D. Huang C. P. (2000). A highly digestible sorghum mutant cultivar exhibits a unique folded structure of endosperm protein bodies. *Proc. Natl. Acad. Sci. U.S.A.* 97 5065–5070 10.1073/pnas.080076297 10792028 PMC25782

[B38] Osborne T. B. (1897). Amount and properties of the proteins of the maize kernel. *J. Am. Chem. Soc.* 19 525–532 10.1021/ja02081a002

[B39] Pastorello E. A. Farioli L. Pravettoni V. Scibilia J. Conti A. Fortunato D. (2009). Maize food allergy: lipid-transfer proteins, endochitinases, and alpha-zein precursor are relevant maize allergens in double-blind placebo-controlled maize-challenge-positive patients. *Anal. Bioanal. Chem.* 395 93–102 10.1007/s00216-009-2945-z 19669736

[B40] Peremyslov V. V. Morgun E. A. Kurth E. G. Makarova K. S. Koonin E. V. Dolja V. V. (2013). Identification of myosin XI receptors in arabidopsis defines a distinct class of transport vesicles. *Plant Cell* 25 3022–3038 10.1105/tpc.113.113704 23995081 PMC3784596

[B41] Poethig R. S. Coe E. H. Johri M. M. (1986). Cell lineage patterns in maize embryogenesis – a clonal analysis. *Dev. Biol.* 117 392–404 10.1016/0012-1606(86)90308-90308

[B42] Schmidt R. J. Burr F. A. Aukerman M. J. Burr B. (1990). Maize regulatory gene opaque-2 encodes a protein with a leucine-zipper motif that binds to zein DNA. *Proc. Natl. Acad. Sci. U.S.A* 87 46–50 10.1073/pnas.87.1.46 2296602 PMC53196

[B43] Segal G. Song R. T. Messing J. (2003). A new opaque variant of maize by a single dominant RNA-interference-inducing transgene. *Genetics* 165 387–397 14504244 10.1093/genetics/165.1.387 PMC1462732

[B44] Shull J. M. Watterson J. J. Kirleis A. W. (1991). Proposed nomenclature for the alcohol-soluble proteins (Kafirins) of *Sorghum bicolor* (L Moench) based on molecular-weight, solubility, and structure. *J. Agric. Food Chem.* 39 83–87 10.1021/Jf00001a015

[B45] Soave C. Salamini F. (1984). Organization and regulation of zein genes in maize endosperm. *Philos. Trans. R. Soc. Lond. Series B Biol. Sci.* 304 341–343 10.1098/rstb.1984.0029

[B46] Song R. Llaca V. Linton E. Messing J. (2001). Sequence, regulation, and evolution of the maize 22-kD alpha zein gene family. *Genome Res.* 11 1817–1825 10.1101/gr.197301 11691845 PMC311139

[B47] Song R. Messing J. (2002). Contiguous genomic DNA sequence comprising the 19-kD zein gene family from maize. *Plant Physiol.* 130 1626–1635 10.1104/pp.012179 12481046 PMC166678

[B48] Torrent M. Alvarez I. Geli M. I. Dalcol I. Ludevid D. (1997). Lysine-rich modified gamma-zeins accumulate in protein bodies of transiently transformed maize endosperms. *Plant Mol. Biol.* 34 139–149 10.1023/A:1005889314967 9177320

[B49] Tsai C. Y. Huber D. M. Warren H. L. (1978). Relationship of kernel sink for N to maize productivity. *Crop Sci.* 18 399–404 10.2135/cropsci1978.0011183X001800030011x

[B50] Vasal S. K. Villegas E. Bjarnason M. Gela W. Goertz P. (1980). “Genetic modifiers and breeding strategies in developing hard endosperm opaque2 materials,” in *Quality Traits of Maize for Grain and Silage Use* edsPollmer W. G. Phillips R. H. (London: Martinus Nijhoff) 37–73

[B51] Wallace J. C. Lopes M. A. Paiva E. Larkins B. A. (1990). New methods for extraction and quantitation of zeins reveal a high content of gamma-zein in modified ppaque-2 maize. *Plant Physiol.* 92 191–196 10.1104/pp.92.1.191 16667246 PMC1062269

[B52] Wang G. F. Wang F. Wang G. Wang F. Zhang X. W. Zhong M. Y. (2012). Opaque1 encodes a myosin XI motor protein that is required for endoplasmic reticulum motility and protein body formation in maize endosperm. *Plant Cell* 24 3447–3462 10.1105/tpc.112.101360 22892319 PMC3462643

[B53] Woo Y. M. Hu D. W. N. Larkins B. A. Jung R. (2001). Genomics analysis of genes expressed in maize endosperm identifies novel seed proteins and clarifies patterns of zein gene expression. *Plant Cell* 13 2297–2317 10.1105/tpc.13.10.2297 11595803 PMC139160

[B54] Wu Y. Holding D. R. Messing J. (2010). Gamma-zein is essential for endosperm modification in quality protein maize. *Proc. Natl. Acad. Sci. U.S.A.* 107 12810–12815 10.1073/pnas.1004721107 20615951 PMC2919962

[B55] Wu Y. Messing J. (2010). RNA interference-mediated change in protein body morphology and seed opacity through loss of different zein proteins. *Plant Physiol.* 153 337–347 10.1104/pp.110.154690 20237020 PMC2862413

[B56] Wu Y. R. Messing J. (2011). Novel genetic selection system for quantitative trait loci of quality protein maize. *Genetics* 188 1019–1022 10.1534/genetics.111.131078 21652527 PMC3176102

[B57] Wu Y. Yuan L. Guo X. Holding D. R. Messing J. (2013). Mutation in the seed storage protein kafirin creates a high-value food trait in sorghum. *Nat. Commun.* 4:2217 10.1038/ncomms3217 23948869

[B58] Xu J. H. Messing J. (2008). Organization of the prolamin gene family provides insight into the evolution of the maize genome and gene duplications in grass species. *Proc. Natl. Acad. Sci. U.S.A* 105 14330–14335 10.1073/pnas.0807026105 18794528 PMC2567223

[B59] Xu J. H. Messing J. (2009). Amplification of prolamin storage protein genes in different subfamilies of the Poaceae. *Theor. Appl. Genet.* 119 1397–1412 10.1007/s00122-009-1143-x 19727653

[B60] Yuan L. Dou Y. Kianian S. F. Zhang C. Holding D. R. (2014). Deletion mutagenesis identifies a haploinsufficient role for γ-zein in opaque2 endosperm modification. *Plant Physiol.* 164 119–130 10.1104/pp.113.230961 24214534 PMC3875793

